# Veterinary Drug Residues in Animal-Derived Foods: Sample Preparation and Analytical Methods

**DOI:** 10.3390/foods10030555

**Published:** 2021-03-07

**Authors:** Bo Wang, Kaizhou Xie, Kiho Lee

**Affiliations:** 1 College of Veterinary Medicine, Yangzhou University, Yangzhou 225009, China; dz120180009@yzu.edu.cn; 2 Joint International Research Laboratory of Agriculture & Agri-Product Safety, Yangzhou University, Yangzhou 225009, China; 3 College of Animal Science and Technology, Yangzhou University, Yangzhou 225009, China; 4 College of Pharmacy, Korea University, Sejong 30019, Korea

**Keywords:** veterinary drugs, animal-derived foods, extraction, detection methods, advanced methods, residues

## Abstract

Veterinary drugs are used to treat livestock and aquatic diseases and thus are introduced into animal-derived foods, endangering consumer health and safety. Antibiotic resistance is rapidly becoming a major worldwide problem, and there has been a steady increase in the number of pathogens that show multi-drug resistance. Illegal and excessive use of veterinary drugs in animals and aquaculture has serious adverse effects on humans and on all other environmental organisms. It is necessary to develop simple extraction methods and fast analytical methods to effectively detect veterinary drug residues in animal-derived foods. This review summarizes the application of various sample extraction techniques and detection and quantification methods for veterinary drug residues reported in the last decade (2010-2020). This review compares the advantages and disadvantages of various extraction techniques and detection methods and describes advanced methods, such as those that use electrochemical biosensors, piezoelectric biosensors, optical biosensors, and molecularly imprinted polymer biosensors. Finally, the future prospects and trends related to extraction methods, detection methods and advanced methods for the analysis of veterinary drug residues in animal-derived foods are summarized.

## 1. Introduction

Veterinary drugs are substances or mixtures used for the prevention, treatment, or diagnosis of animal diseases or for purposeful regulation of animal physiological functions [1]. During recent decades, different types of veterinary drugs have been used in animals and aquaculture for high-yield production. Veterinary drugs have indeed been used to treat some diseases of farm animals, such as poultry, pigs, and cattle, but long-term use of veterinary drugs has caused drug resistance in animals [1]. In the process of aquaculture, the use of veterinary drugs can easily lead to water environmental pollution and affect the safety of drinking water. Excessive and illegal use of some veterinary drugs poses a severe threat to human health and causes environmental pollution, leading to the death of some animals, plants, and microorganisms [2,3,4]. Veterinary drug residues have become a hot issue, and various countries are advocating the reduced use of antibiotics and development of new alternative veterinary drugs to minimize the harm caused by veterinary drug residues.

**Figure 1:**
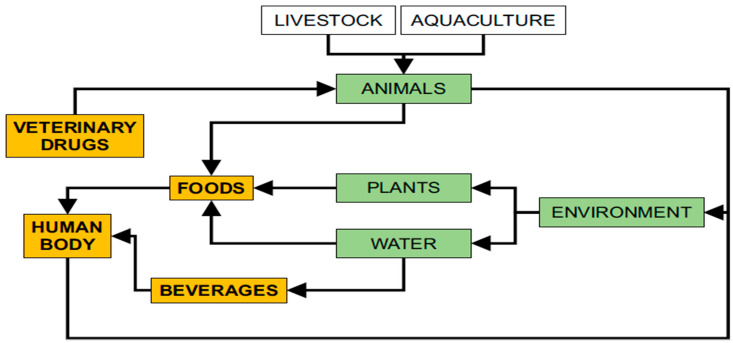
A series of processes involving veterinary drug residues in the human body.

To date, veterinary drugs are still used to treat diseases in farmed animals and in aquaculture. Veterinary drugs are introduced to the animal’s body through three routes—via animal feed, oral administration, or injection—and most are added to the feed. Veterinary drugs are metabolized by animals, and some of the drugs remain in the animal body, while others enter the environment through excreta. In aquaculture, veterinary drugs usually enter fish, shrimp, and crabs as well as other aquatic products and rivers. The veterinary drugs in these excretions and in rivers are absorbed by vegetables and by fruit trees. Humans drink water and eat vegetables and fruit containing veterinary drugs. These drugs re-enter the body and seriously endanger human health. We summarized the information on veterinary drug residues in the environment and animal-derived foods in Figure 1. To protect the health and safety of consumers, the European Union (EU), United States, China, and other countries have established maximum residue limits (MRLs) for veterinary drugs in animal-derived foods [5,6,7].

The use of veterinary drugs has rapidly increased, mainly in farm animal breeding and aquaculture, allowing high-yield and high-quality production of animal-derived foods [8]. However, excessive use of these drugs leads to harmful residues, including metabolites and original drugs, which can endanger animal and human health. Qualitative and quantitative analyses of these drugs are needed to ensure the safety of animal-derived food and combat illegal and excessive use of veterinary drugs in the animal breeding industry. Therefore, many detection techniques have been developed to detect veterinary drug residues in animal-derived foods. Currently, the classic analysis methods commonly used for veterinary drugs include enzyme-linked immunosorbent assay (ELISA) [9], capillary electrophoresis (CE) [10], liquid chromatography (LC) [11] and gas chromatography (GC) [12]. Generally, these methods have high sensitivity and selectivity in the detection of animal-derived foods. Due to the complexity of the matrix, a sample pre-treatment process is usually required before instrument testing. The methods for extracting veterinary drugs from animal-derived foods mainly include liquid-liquid extraction (LLE) [13]; solid-phase extraction (SPE) [14]; accelerated solvent extraction (ASE) [15]; quick, easy, cheap, effective, rugged and safe (QuEChERS) extraction [16]; matrix solid-phase dispersion (MSPD) extraction [17]; ultrasound-assisted extraction (UAE) [18] and solid-phase microextraction (SPME) [19]. However, traditional detection methods combined with these sample pre-processing techniques are limited by disadvantages such as cumbersome operations, large time costs, and expensive instruments. The second approach is to detect veterinary drug residues in animal-derived foods via advanced devices based on sensing principles, including electrochemical biosensors, piezoelectric biosensors, optical biosensors, and molecularly imprinted polymer (MIP) biosensors [20,21]. Compared with traditional detection methods, advanced methods have the advantages of being fast, simple, low cost, highly sensitive, and highly selective, but the limit of detection of the sensor cannot reach the same level, and the quantitative accuracy is not as good as that of traditional detection methods.

The detection of veterinary drug residues in animal-derived foods is very important. Therefore, this article briefly describes the classification, use and physicochemical characteristics of veterinary drugs, sample preparation techniques, comparison of traditional detection methods and introduction of advanced methods in the past decade (2010–2020). The purposes of this review are to provide insight into the research progress of the detection methods of veterinary drug residues in animal-derived foods and to compare the advantages and disadvantages of different detection methods so that researchers can choose the appropriate method for further research.

## 2. Veterinary Drugs: Classification, Use and Physicochemical Characteristics

**Figure 2:**
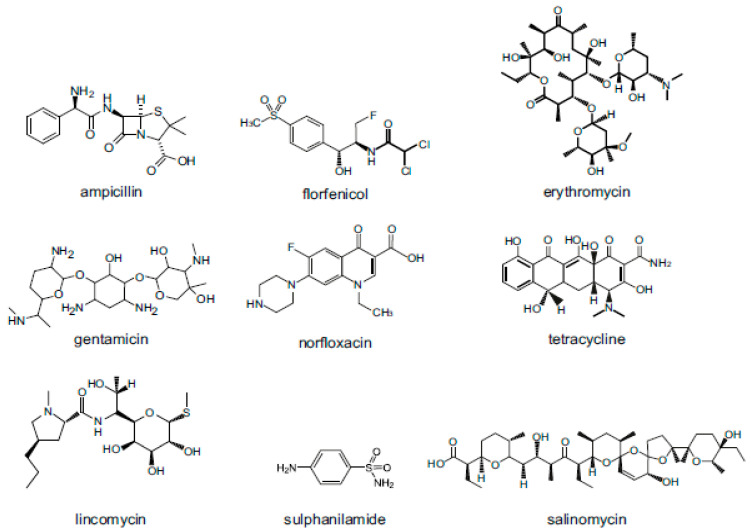
Structures of representative compounds from each class of antimicrobials used as veterinary drugs.

Veterinary drugs are classified according to their source, use, and chemical structure and can be classified as natural drugs, semi-synthetic drugs, and synthetic drugs. According to their use, veterinary drugs can be roughly classified into four categories: general disease control drugs, infectious disease control drugs, internal and external parasitic disease control drugs, and growth-promoting drugs. Generally, researchers classify compounds according to their structure and function, such as antimicrobials, corticosteroids, analgesics, anti-parasitics and hormones. This article will introduce the use, antibacterial mechanism, and toxicity of nine types of veterinary drugs: penicillins (PCNs), amphenicols (APs), macrolides (MACs), aminoglycosides (AGs), quinolones (Qs), tetracyclines (TCs), lincosamides (LAs), sulphonamides (SAs) and coccidiostats (COCs). The structures of representative compounds from each class of drugs studied are shown in (Figure 2).

PCNs are a group of antibiotics, including natural PCNs (penicillin G, K, N, O, and V), β-lactamase-resistant PCNs (methicillin, oxacillin, and cloxacillin), aminopenicillins (ampicillin (AMP), amoxicillin, and pivampicillin), carboxypenicillins (carbenicillin, ticarcillin, and temocillin), ureidopenicillins (mezlocillin, azlocillin, and piperacillin) and β-lactamase inhibitors (clavulanic acid, sulbactam, and tazobactam). The antibacterial mechanism of PCNs involves inhibition of cell wall synthesis by inhibiting the enzyme activity required for cross-linking of peptidoglycans in the bacterial cell wall, leading to cell lysis and death [22]. Natural PCNs cannot tolerate the enzymes produced by drug-resistant strains (such as drug-resistant *Staphylococcus aureus*) and are easily destroyed by these enzymes. In addition, the antibacterial spectrum of these drugs is relatively narrow, and they are mainly effective against gram-positive bacteria. PCNs can kill bacteria by interfering with the synthesis of bacterial cell walls [22]. PCNs have low toxicity, but a small number of people are allergic to these drugs, exhibiting conditions such as skin rashes, drug fever, and asthma [23].

APs are a class of broad-spectrum antibiotics, including mainly chloramphenicol (CAP), thiamphenicol (TAP) and florfenicol (FF). The antibacterial mechanism of APs involves inhibition of bacterial protein biosynthesis via control of the peptidyl transferase of bacterial ribosomes. APs are antibiotics that are useful for the treatment of several bacterial infections, which have an effect on both gram-positive bacteria and gram-negative bacteria [24]. CAP can cause serious side effects (inducing aplastic anemia), so it is listed as a banned drug by the EU [5]. TAP is a derivative of CAP but is less toxic than CAP, causing diseases such as aplastic anemia, bone marrow suppression and liver toxicity [25]. FF, as the third-generation product of CAP, has low toxicity and is still used to treat animal diseases.

MAC is a general term for a class of antibacterial drugs with 12–16 carbon lactone rings in the molecular structure, mainly including erythromycin and its ester derivatives (azithromycin and roxithromycin), clarithromycin and telithromycin. The mechanism of action of MACs involves inhibition of bacterial protein biosynthesis by preventing peptidyl transferase from adding the growth peptide linked to tRNA to the next amino acid (similar to chloramphenicol) and inhibiting ribosomal translation [26,27]. MACs can be used to treat infections caused by gram-positive bacteria, a limited number of gram-negative bacteria, and some respiratory and soft-tissue infections [28]. The toxicology of MAC antibiotics includes mainly gastrointestinal symptoms, liver toxicity, cardiotoxicity, and allergic reactions [29].

AGs are so named because their molecular structure has an amino cyclic alcohol and one or more amino sugar molecules, which are connected by glycosidic bonds to form glycosides [30]. Kanamycin A, amikacin, tobramycin, dibekacin, gentamicin, sisomicin, netilmicin, neomycin B, neomycin C, neomycin E, streptomycin, and plazomicin are all AG antibiotics. As antibacterial agents, AGs act on ribosomes in bacteria, inhibit protein synthesis, and destroy the integrity of bacterial cell membranes [30]. AGs show bactericidal activity against gram-negative aerobes and some anaerobic bacteria but have no resistance to gram-positive and anaerobic gram-negative bacteria [31]. The main adverse reactions of AGs are nephrotoxicity and ototoxicity, especially in children and the elderly [32,33].

Qs (e.g., pipemidic acid, oxolinic acid, and cinoxacin) and their synthetic fluoride-containing derivatives, fluoroquinolones (FQs) (e.g., ciprofloxacin and ofloxacin), are members of a large group of broad-spectrum bactericidals that share a bicyclic core structure related to the substance 4-quinolone or 4-fluoroquinolone [34,35]. The target enzymes of Qs are bacterial DNA gyrase (gyrase) and topoisomerase IV. In most gram-negative bacteria, DNA gyrase is the main target enzyme for Qs. In most gram-positive bacteria, Qs mainly inhibit bacterial topoisomerase IV, which is a helicase that can release the entangled chromosomes of the progeny during DNA replication. Nearly all quinolone antibiotics in use are FQs, which contain a fluorine atom in their chemical structure and are effective against both gram-negative and gram-positive bacteria [36,37]. Qs and FQs can cause allergic reactions, affect cartilage development, cause liver damage, and have other adverse effects, especially in juvenile animals, and in children, these drugs can cause arthropathy [38].

TCs (e.g., chlortetracycline, oxytetracycline, and tetracycline) are a group of broad-spectrum antibiotic compounds with a common basic structure that can be directly isolated from several species of *Streptomyces*, or they can be obtained by semi-synthesis [39]. The mechanism of action of TCs is that the drug can specifically bind to the A position of the 30S subunit of the bacterial ribosome to prevent the connection of aminoacyl-tRNA at this position, thereby inhibiting the growth of peptide chains and affecting the synthesis of bacterial proteins [40]. TCs have a broad antibacterial spectrum and have antibacterial effects on gram-positive and gram-negative bacteria, rickettsiae, spirochetes, mycoplasma, chlamydia, and certain protozoa [41]. TCs can cause liver damage, exhibit nephrotoxicity, are not conducive to bone and tooth growth, and cause gastrointestinal reactions [42,43].

LAs are a class of powerful, narrow-spectrum antibacterial drugs produced by *Streptomyces* and include lincomycin, clindamycin, and pirlimycin [44]. As antibacterial agents, LAs prevent bacterial replication by interfering with protein synthesis and have an antibacterial effect on most gram-positive bacteria and some anaerobic gram-negative bacteria [45]. LAs are usually used clinically as alternative antibiotics for patients allergic to penicillin. In veterinary microbiology, LAs are used as first-line antibiotics to combat skin infections [46]. The toxicology of LAs causes gastrointestinal dysfunction, allergic reactions, leukopenia and thrombocytopenia [47].

SAs (e.g., sulphanilamide, acetohexamide, and ethoxzolamide) are a group of synthetic drugs containing sulphonamide chemical groups and are the first widely used antibacterial agents to be systematically used [48]. According to clinical use, sulpha drugs can be divided into three categories: sulpha drugs that are easily absorbed in the intestine, sulpha drugs that are difficult to absorb in the intestine, and sulpha drugs for external use. As antibacterial agents, SAs act as competitive inhibitors of dihydropterin synthase (DHPS, an enzyme involved in folic acid synthesis), which can inhibit the growth and reproduction of bacteria [49]. SAs are used to treat allergies and coughs, as well as antifungal and antimalarial functions. The toxicology of SAs mainly includes allergic reactions, kidney damage, hematopoietic effects, and central nervous system and gastrointestinal reactions [50,51].

COCs used as anti-coccidials come from several different drug classes, including nitroimidazoles, ionophores, triazines, benzamides, carbanilides, quinolone derivatives, and other anti-coccidials [52]. COCs have four possible mechanisms of action: they affect ion transport through cell membranes, affect coenzyme absorption and synthesis, affect mitochondrial function and act on plastids. COCs have effects on both gram-positive bacteria and gram-negative bacteria, which are widely used to prevent poultry breeding [53,54]. Since COCs are generally used for a long time, residues in meat and eggs are inevitable, often affecting product quality and human health. Therefore, it is necessary to strictly enforce the withdrawal period for COCs.

After these drugs enter the animal body, they undergo physical and chemical reactions and finally remain in the meat, milk, eggs and animal tissue food as the original drug or metabolites. In addition, the original drug or metabolites are discharged into the environment through excrement. The study of drug metabolism of antibiotics in different animals involves pharmacokinetic studies. A well-developed detection method is conducive to the study of pharmacokinetics and drug elimination rules to determine the withdrawal period and time to market.

## 3. Extraction Methods

Generally, the detection of veterinary drug residues in animal-derived foods requires sample pre-processing, instrumentation method establishment and data analysis to evaluate the stability, precision, and sensitivity of the established method. Animal-derived food samples have a complex matrix and many endogenous interfering substances, making it impossible to directly detect veterinary drug residues. Before sample testing, sample pre-treatment steps such as extraction, purification, evaporation, concentration, and reconstitution are usually required.

### 3.1. Liquid-Liquid Extraction (LLE)

LLE is a traditional sample pre-treatment method that includes solvent extraction and ultrasonic vibration-mediated extraction. Different extraction reagents are used to extract veterinary drug residues from animal-derived foods, including acetonitrile (ACN) [55,56], ethylenediaminetetraacetic acid disodium salt (EDTA)-succinate [57], 0.1% formic acid in aqueous solution of EDTA 0.1% (*w/v*)–ACN–methanol (MeOH) (1:1:1, *v/v*) [58], acidified methanol 1% HCOOH [59] and ethyl acetate-ACN-ammonium hydroxide (49:49:2, *v/v*) [60].

Recently, Xie et al. [60] reported an LLE method combined with a high-performance liquid chromatography-tandem triple quadrupole mass spectrometry (HPLC-MS/MS) analytical system to detect CAP, TAP, FF, and FF amine in egg samples. The limit of detection (LOD) and limit of quantification (LOQ) were 0.04–0.5 μg/kg and 0.1–1.5 μg/kg, respectively, and the extraction recovery rate was 90.84–108.23%, with relative standard deviations (RSDs) of less than 9.61% and correlation coefficients (*R^2^*) exceeding 0.9994. The developed method has shown good sensitivity and recovery rates. Dasenaki and Thomaidis [58] extracted 115 veterinary drugs from milk powder, butter, fish tissue and eggs using the LLE method prior to LC-MS/MS. The LLE-LC-MS/MS method showed low LODs and LOQs ranging from 0.008 μg/kg to 3.15 μg/kg with a correlation R^2^ value exceeding 0.99, and the RSD values obtained were less than 18%. In another study, conducted by Tang et al. [55], an efficient, fast, and convenient method based on LLE and ultra-performance liquid chromatography (UPLC)–MS/MS was developed for the determination of 23 veterinary drugs in milk with LOD values from 0.1–2.5 ng/mL.

Chung and Lam [56] developed ultra-performance hydrophilic interaction LC (HILIC) and reversed-phase LC (RPLC) coupled to an MS/MS spectrometer for the simultaneous detection of 15-class veterinary drugs in milk, egg, and meat. The proposed HILIC-MS/MS and RPLC-MS/MS methods coupled with LLE are simple and efficient extraction and detection techniques that can detect recovery values ranging between 70% and 120% in milk, egg and meat samples with good precision and linearity. The LLE method has been used to extract veterinary drug residues from animal-derived foods for nearly a decade. The method is simple in operation but has disadvantages such as high reagent consumption, time consumption and chance of manual error. Moreover, toxic organic solvents are usually used in the LLE extraction process, as researchers must take protective measures to avoid physical harm.

### 3.2. Solid-Phase Extraction (SPE)

SPE is a fast and selective sample preparation and purification technique that is performed before chromatographic analysis. SPE technology allows sample purification, recovery, and concentration for precise quantitative analysis. The principle underlying the selectivity of SPE is similar to that of LC. Compared with the traditional LLE method, SPE can improve the recovery rate of the analyte, separate the analyte from the interfering components more effectively, and reduce sample pre-treatment processing, making it simple in operation and saving time and effort [61]. Common SPE cartridges include CNWBOND LC-C18 SPE cartridges [61], EVOLUTE ABN SPE cartridges [62], hydrophilic-lipophilic balance (HLB) SPE cartridges [63,64,65,66,67], and hybrid SPE cartridges [68], which are used to extract veterinary medicines from meat, milk, eggs, honey, fish, shrimp, eel, and animal tissues.

Recently, Wang et al. [61] applied CNWBOND LC-C18 SPE cartridges to extract eight kinds of COCs (robenidine, halofuginone, lasalocid, monensin, nigericin, salinomycin, narasin, and maduramicin) from egg samples. The CNWBOND LC-C18 SPE cartridge has unique selectivity, and the long carbon chain also exhibits strong non-polarity. Because of its relatively low carbon content, it is more suitable for retaining polar compounds or non-polar compounds that are too large. HPLC-MS/MS and UPLC-MS/MS methods were used to determine and quantify these compounds. The recoveries of the two methods were more than 71.7%, and the LOD values (0.16–0.52 μg/kg) were lower than the MRLs of these drugs. Another study conducted by Kaufmann et al. [62] analyzed more than 100 different veterinary dugs from various food matrices (muscle, kidney, liver, fish, and honey). This study compared OASIS HLB SPE cartridges and ABN SPE cartridges, and the results showed that ABN SPE cartridges achieved good extraction recovery. SPE technology combines UPLC with high-resolution mass spectrometry (HRMS) to quantitatively detect these analytes, and the LOD (1 μg/kg) of these analytes is much lower than the value set by the EU. The development of this method has greatly improved the detection efficiency, and more than one hundred drugs can be measured simultaneously.

Dasenaki et al. [63] used HLB SPE cartridges combined with UPLC quadrupole time-of-flight mass spectrometry (QTOF-MS) to extract and detect 143 veterinary drugs from milk and fish tissue. The QTOF-MS instrument can simultaneously detect more than one hundred compounds and can accurately analyze these compounds quantitatively and qualitatively. This study uses the SPE method to effectively extract milk samples, which can reduce matrix effects and enhance sensitivity. A study by Piatkowska et al. [68] used zirconium-coated silica as an SPE sorbent to extract 13 classes of veterinary drugs. The obtained recoveries of egg samples were more than 75% among all veterinary drugs, and the correlation (*R^2^*) value was more than or equal to 0.99. The RSDs of the repeatability and reproducibility were 1.6–15.9% and 2.6–15%, respectively. The choice of SPE cartridge is one of the important factors that affect the extraction recovery from animal-derived food samples. As shown in Table 1, this article compares the efficacy of different cartridges for veterinary drugs in animal-derived foods. CNWBOND LC-C18 SPE cartridges can effectively extract eight COCs from eggs, and EVOLUTE ABN SPE cartridges, OASIS HLB SPE cartridges and Hybrid SPE cartridges can simultaneously extract multiple residues of veterinary drugs from animal-derived foods. Because of the good extraction efficiency of OASIS HLB SPE cartridges, they are widely used for veterinary drug residues in animal-derived foods [12,63,64,65,66,67]. The SPE method is widely used in the extraction of veterinary drug residues from animal-derived foods. Efficient and simple extraction technology is conducive to the extraction of multiple residues. In addition, LLE and SPE are often used in combination to better enrich and purify veterinary drugs in animal-derived food samples.

### 3.3. Accelerated Solvent Extraction (ASE)

**Figure 3:**
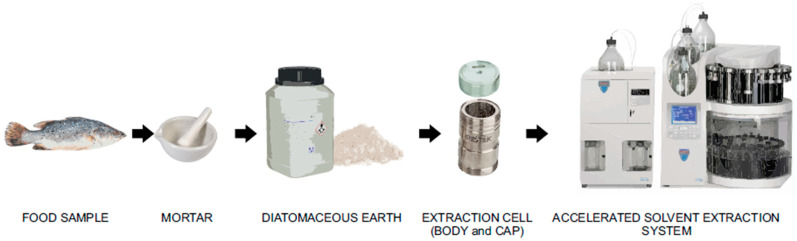
Flowchart of accelerated solvent extraction procedures of animal-derived food samples.

ASE is an automated method for extraction with organic solvents under conditions of elevated temperature and pressure. Richter et al. [69] introduced ASE as a new extraction procedure that uses organic solvents to extract solids or semi-solids at higher pressures (500–3000 psi) and higher temperatures (50–200 °C). The advantages of the ASE method are the small amounts of organic solvents, high speed, low matrix effect, high recovery rate and good reproducibility, and it appears as the recommended method 3545 in update III of the US EPA SW-846 methods [70]. The ASE method is widely used to extract veterinary drug residues from animal-derived foods, and a brief flowchart of ASE sample preparation is shown in Figure 3. The animal-derived food samples are placed into a mortar and added to diatomaceous earth for grinding. After being fully ground, the sample is filled into a 22 mL stainless steel extraction cell, and then the lid is closed. The cell is placed on the ASE350 instrument, and the sample processing program is set.

Wang et al. [12] have developed a fast and sensitive ASE method coupled with gas chromatography-tandem mass spectrometry (GC-MS/MS) for the detection of spectinomycin and lincomycin in poultry eggs. This study used an ASE350 instrument and an Oasis PRiME HLB SPE cartridge to extract and purify egg poultry samples. The proposed method successfully detected spectinomycin and lincomycin with LODs and LOQs ranging between 2.3–4.3 μg/kg and 5.6–9.5 μg/kg, respectively. This method has a good correlation coefficient (*R^2^* ≥ 0.9991), recovery (80.0–95.7%) and precision (RSDs, 1.0–3.4%). Compared with the ASE-HPLC-MS/MS method [11], the ASE-SPE-GC-MS/MS method involves sample preparation steps that are complicated and require solid-phase extraction, which greatly increases the processing time. Tao et al. [71] used the ASE method to extract 17 MAC and avermectin residues in swine and bovine tissues (muscle, kidney, and liver) at 60 °C and 1500 psi for 10 min (static time) in two cycles, with ACN/methanol (1/1, *v/v*) as the extractant. After sample preparation, this study used the LC-MS/MS method to detect these analytes. The recoveries of the samples were all higher than 75%, and the LOD values were all lower than 0.55 g/kg. This study shows that ASE technology can extract multiple residues, which has advantages such as high speed, low consumption of reagents, and batch processing of samples.

Yu et al. [72] reported an ASE-HPLC-UV method for the detection of seven TCs in pig, chicken, and cattle tissues (muscle and liver). The LOD and LOQ values were lower than 10 μg/kg and 15 μg/kg, respectively. Within the range of concentrations used, the sample recovery was 75.0–104.9%, and the RSD was lower than 10%. A novel method was proposed by Wang et al. [11], who used an ASE350 instrument for sample pretreatment with methanol-ammonium hydroxide-ultrapure water (97:2:1, *v/v*) as the extractant. This study used HPLC-MS/MS to detect CAP, TAP, FF, and FF amine in poultry eggs. ASE extracts APs from poultry eggs to obtain a good extraction recovery rate, and the detection sensitivity of the method is relatively high (LOD values are all lower than 0.5 μg/kg). Compared with LLE and SPE methods, ASE has the advantages of simple operation, high speed, and batch processing of samples, greatly improving efficiency and saving time. With the development of sample preparation technology, the automated ASE method is worthy of promotion for the extraction of veterinary drug residues from animal-derived foods.

### 3.4. Quick, Easy, Cheap, Effective, Rugged and Safe (QuEChERS) Extraction

The steps of the QuEChERS method can be simply summarized as follows: (1) crushing of the sample; (2) single-solvent (acetonitrile) extraction and separation; (3) addition of MgSO_4_ and other salts to remove water; (4) addition of adsorbent to remove impurities; and (5) GC-MS and LC-MS analysis of the supernatant. The QuEChERS extraction method is widely used for multi-class or multi-residue analysis of different types of veterinary drugs in animal-derived foods. The principle of QuEChERS is similar to that of HPLC and SPE. It uses the interaction between the adsorbent filler and the impurities in the matrix to adsorb impurities, thereby achieving impurity removal and purification. Anastassiades et al. [73] first proposed the QuEChERS method, which can extract both polar and non-polar compounds.

Recently, Xu et al. [74] used the QuEChERS method to extract veterinary drugs, pesticides, and mycotoxins from egg samples. The modified QuEChERS method used magnetic multiwalled carbon nanotubes (Fe_3_O_4_-MWCNTs) as the adsorbent and achieved faster separation of the adsorbent by using an external magnet. Among multiple residues present in egg samples, 48 veterinary drugs, 13 pesticides and 13 mycotoxins were detected by using UPLC-MS/MS analytical systems with LOQs ranging from 0.1 μg/kg to 17.3 μg/kg. The obtained recoveries were 60.5–114.6% at three fortified levels with RSDs of less than 20%. Arias et al. [75] used the inexpensive and green material chitosan as an adsorbent based on the QuEChERS method to extract 7 types of veterinary drug residues from milk. Chitosan was obtained from shrimp shell waste, and the optimized QuEChERS method combined with LC-MS/MS was used to quantitatively analyse the multiple residues of veterinary drugs from milk samples with good selectivity, accuracy, and precision. The LOQs ranged between 1 and 50 μg/kg, and recoveries ranged between 62 and 125%, with an RSD <20%.

A modified QuEChERS procedure combined with the UPLC-QTOF-MS analysis method was used to detect 90 veterinary drugs in royal jelly [76]. In this method, modification of the QuEChERS procedure was performed for acid hydrolysis and protein precipitation (citric acid and Na_2_HPO_4_), including modification of extraction reagents (acetic acid-acidified acetonitrile), partitioning salts (sodium chloride‒anhydrous sodium sulfate) and MSPD sorbents (NH_2_ cartridges). This method achieved good correlation (*R^2^*, 0.9921–0.9999), recovery (70.2–120.1%), precision (1.77–9.90%), repeatability (3.01–11.6%) and reproducibility (5.97–14.9%). Another study based on the application of the QuEChERS method was conducted by Shin et al. [77] and detected 50 veterinary drug residues in fishery products. For the QuEChERS method, a dispersive SPE (d-SPE) method using primary secondary amine (PSA) and octadecylsilane (C18) absorbents was selected to prevent matrix interference during mass spectrometry analysis. The recoveries of 50 veterinary drugs in fishery products were 68.1–111%, the RSD was <15%, and the LODs and LOQs were <5 and <10 μg/kg, respectively. The studies [78,79,80,81] indicate that QuEChERS is a simple, fast, environmentally friendly, and economical method that is suitable for the analysis of multiple residues of veterinary drugs in animal-derived food.

### 3.5. Matrix Solid-Phase Dispersion (MSPD) Extraction

Barker et al. [82] first proposed the MSPD method as a rapid sample processing technique suitable for extracting multiple drug residues from a single sample. Compared with modern extraction technology that uses high pressure and high temperature (ASE), MSPD performs the extraction process under ambient conditions and does not require any special laboratory equipment. It has advantages over conventional techniques, requiring only a few simple steps to extract a small number of samples and solvents [83]. Based on these advantages, the MSPD method is widely used in the extraction of multiple veterinary drug residues from animal-derived foods.

Wang et al. [84] reported a novel, fast and simple mixed-template molecularly imprinted polymer (MMIP)-MSPD extraction method combined with the UPLC-photodiode array (PDA) detector analysis method to detect 8 FQs, 8 SAs and 4 TCs in pork. The extraction procedure is based on MSPD using MMIP as a dispersant and methanol/acetic acid (9:1, *v/v*) as the eluent. The sample recoveries with this method exceeded 92%, and the LODs of the 20 drugs in pork were 0.5–3.0 μg/kg, which shows that this method has good sensitivity and selectivity. Another MSPD method was developed by Shen et al. [85], using HLB material as the sorbent and a pipette tip (PT) as the cartridge, to extract and purify 14 SAs from fish tissue. After the sample was processed by PT-MSPD, the eluate was analyzed by LC-MS/MS. This method is fast (5 min for PT-MSPD and 8 min for LC-MS/MS), with good recovery (70.6–95.5%), precision (1.4–10.3%), sensitivity (LOD, 2.3–16.4 μg/kg) and selectivity (LOQ, 6.9–54.7 μg/kg). Compared with the traditional MSPD method, the PT-MSPD method has better recovery and precision. Pan et al. [17] and Tao et al. [86] demonstrated that octadecylsilyl-derivatized silica (C18) can separate CAP, TAP and FF from fish and shrimp. Moreover, da Silva et al. [87] reported a new method that uses electrical (E)-MSPD for the extraction and clean-up of 7 FQs in bovine milk. Florisil, silica gel, or C18 can be used for sample dispersion and extraction of tetracycline, oxytetracycline and doxycycline, as previously reported by Mu et al. [88]. The blending of pork muscle samples with the Oasis HLB adsorbent has been used in the MSPD method [89]. MSPD extraction technology helps simplify the sample preparation process. Therefore, it is considered to be a simple, fast, cost-effective, and environmentally friendly method that is suitable for applications in food, animal tissues, plant material and environmental samples [90,91]. This article describes in detail the application and research progress of the LLE, SPE, ASE, QuEChERS and MSPD methods in veterinary drug residues in animal-derived foods. In addition, this article compares the advantages and disadvantages of these five extraction methods, as shown in (Table 2).

### 3.6. Other Extraction Methods

LLE, SPE, ASE, QuEChERS, MSPD and other types of extraction methods are used for the determination of veterinary drug residues in animal-derived foods, as summarized in Table 3. These include UAE [18], gel permeation chromatography (GPC) [92], turbulent flow chromatography (TFC) [93], fabric phase sorptive extraction (FPSE) [94], SPME [19,95], solid-liquid extraction (SLE) [96], liquid-phase microextraction (LPME) [97] and dispersive liquid–liquid microextraction (DLLME) [98].

## 4. Analytical Methods for Detection

After the sample preparation step, the next step is to detect these analytes in animal-derived foods through the use of instruments. In the past decade, many techniques were developed for the detection of veterinary drug residues in animal-derived food samples. Due to the wide variety of veterinary drugs, traditional thin-layer chromatography, LC and GC analysis methods combined with different types of detectors are used to detect and analyse veterinary drugs in animal-derived foods. In addition, traditional analytical methods, including ELISA, CE and micellar electrokinetic capillary chromatography (MEKC), and advanced devices, including electrochemical biosensors, piezoelectric biosensors, optical biosensors, and MIP biosensors, are used to analyse veterinary drugs in animal-derived foods.

### 4.1. Liquid Chromatography (LC)

LC is a common, efficient, and rapid chromatographic method to detect veterinary drugs in animal-derived foods. The key to LC separation is to select a suitable chromatographic column and optimize the composition of the mobile phase and the elution procedure. The LC method has wide applicability and can be used for most veterinary drugs. Generally, analyses of veterinary drugs are conducted by LC coupled with specific detectors, such as fluorescence detectors (FLDs), diode array detectors (DADs), ultraviolet detectors (UVDs) and evaporative light scattering detectors (ELSDs). To date, many methods based on LC combined with various detectors to detect veterinary drugs in animal-derived foods have been reported, including LC-FLD [99,100], LC-DAD [94,96,97,101,102], LC-UVD [103,104,105,106,107,108,109,110] and LC-ELSD [111]. At present, the pairing of LC with mass spectrometer detectors (MS and tandem MS) has been widely used in the analysis of veterinary drugs in animal-derived foods [112,113,114].

Different types of detectors combined with LC are used to detect the same type or different types of veterinary drugs and have their own advantages and disadvantages [11,99]. FLDs are highly sensitive and selective detectors that can detect only compounds that produce fluorescence. DADs and UVDs are mainly used to detect veterinary drugs containing ultraviolet absorbing groups, and they have the advantages of high sensitivity, low noise and wide linear range. ELSDs can detect any sample with lower volatility than the mobile phase. Due to this limitation, it has rarely been used in the detection of veterinary drug residues in animal-derived foods in the past decade. Compared with FLDs, DADs, UVDs and ELSDs, MS detectors can simultaneously detect more than 100 veterinary drugs in animal-derived foods. In addition, MS detectors have high recovery, high selectivity, good reproducibility, and low interference. Moreover, the use of tandem MS improves sensitivity and plays an important role in confirming false positives [115,116]. The rapid development of MS detectors, such as triple quadrupole-MS, TOF-MS, QTOF-MS and Orbitrap-HRMS instruments, has greatly improved the efficiency of detecting veterinary drugs in animal-derived foods. The LC-MS/MS method is commonly used to detect veterinary drug residues in animal-derived foods.

### 4.2. Gas Chromatography (GC)

GC is a commonly used chromatographic technique that mainly uses differences in the boiling point, polarity, and adsorption properties of compounds to separate mixtures. For the analysis of veterinary drug residues in animal-derived foods, GC instruments are usually connected to classic detectors, mainly including nitrogen-phosphorus detectors (NPDs), electron capture detectors (ECDs) and MS detectors [117,118]. To date, GC-MS and GC-MS/MS methods are the most commonly used methods to detect veterinary drugs in animal-derived foods. Compared with NPDs and ECDs, MS or MS/MS has good recovery, precision, and reproducibility and can confirm false positives. Generally, derivatization reactions are required for the detection of veterinary drugs by GC [12,119]. GC usually requires the selection of specific capillary columns to separate the veterinary drugs in the sample, while optimization of the mobile phase, as in the LC method, is not required.

GC instruments are relatively expensive, and researchers usually need professional training to operate the instruments. GC is widely used for analysis of pesticides, and GC-MS/MS methods are being gradually developed for research on veterinary drugs. The main reason is that mass spectrum information for some veterinary drugs in the GC mass spectrum library is lacking. Table 4 summarizes some published LC and GC methods for the detection of veterinary drugs in animal-derived foods.

### 4.3. Enzyme-Linked Immunosorbent Assay (ELISA)

ELISA is an ultramicro-experimental detection technology with high sensitivity and specificity established by combining modern detection methods with immune technology. ELISA has the advantages of easy operation, convenience, high efficiency, strong specificity, and low detection cost and is widely used in the detection of veterinary drug residues in animal-derived foods [130,131,132,133,134,135,136,137,138,139]. Samsidar et al. [20] introduced two forms ELISA: direct competitive (dc)-ELISA and indirect competitive (ic)-ELISA, of which the ic-ELISA method is more advanced. In the past decade, this reliable high-throughput immunoassay has been widely used to determine various veterinary drugs in animal-derived foods. The basic principle of ELISA is to combine a specific antigen-antibody immunological reaction with an enzymatic catalytic reaction and to display the primary immune response with amplification of the enzymatic reaction. This method can detect both antigen and antibody.

Several ELISA methods for the detection of veterinary drugs have been reported. An et al. [130] applied the ic-ELISA method to detect FF and TAP in edible animal tissue and feed. The recovery, precision and LODs of FF and TAP in animal tissues are 80.6–105.5%, 3.5–14.1% and 0.07–0.14 μg/kg, respectively. These values show that the ic-ELISA method successfully detected FF and TAP in animal tissues. Zhou et al. [134] reported a novel, convenient and efficient ic-ELISA method to detect 20 SA residues in edible animal tissues. The LODs of SAs in animal tissues were 1.5–22.3 μg/kg, and the recoveries were 70.6–121.0%, with less than 24.1% RSD. A comparative study of ELISA and HPLC-UVD methods was performed by Bahmani et al. [135] for analysis of TCs in a multiresidue sample. For the ELISA method, the recovery of 4 TCs was 71.9–100.0%, with an RSD lower than 10%, and the LODs and LOQs of meat and raw milk samples were 3.7–9 μg/kg and 9–27 μg/L, respectively. This study also used the HPLC-UVD method to confirm the analysis of TC residues in meat and raw milk samples. The recovery and precision of the ELISA method are lower than those of the HPLC-UVD method, but the sample preparation steps are simple, and the recovery and precision of the ELISA method meet the EU method parameter requirements [140]. The ELISA method simplifies the sample preparation steps, greatly shortens the entire experimental duration, and does not require expensive equipment. Due to its advantages of being inexpensive, selective, and efficient, the ELISA method is still used for the screening of veterinary drug residues in animal-derived foods.

### 4.4. Capillary Electrophoresis (CE)

CE is a new type of liquid-phase separation technology that uses capillaries as the separation channel and a high-voltage direct current electric field as the driving force. CE is an efficient, fast, and economical automated separation technology with the advantages of low reagent consumption, small sample injection volume and high separation efficiency. CE has certain limitations in sample preparation ability, sensitivity, and separation reproducibility. The reasons for these limitations are the small injection volume, small capillary diameter, and electroosmotic changes due to sample composition. Due to the low sensitivity caused by the small injection volume, CE has been combined with some high-sensitivity detectors, including UVDs [141,142,143], laser-induced fluorescence (LIF) detectors [144], electrochemiluminescence (ECL) detectors [145], DADs [146], MS detectors [147,148] and chemiluminescence (CL) detectors [149].

Recently, Yang et al. [150] developed a pressure-assisted electrokinetic injection (PAEKI) method coupled with capillary zone electrophoresis (CZE) for the determination of 6 SAs in different animal husbandry products. Briefly, an on-line PAEKI method for the simultaneous enrichment of six SAs was used to increase the sensitivity of CZE. The LODs and LOQs ranged from 1.8–63.8 μg/L and 6.1–182.6 μg/L, respectively, in milk, pork and egg samples. Moreno-González et al. [148] reported the use of MIPs as SPE sorbents (MISPE) to extract 9 AGs from honey samples via CZE-MS/MS determination. In this study, the MISPE extraction method and an MS/MS detector were used, which greatly improved the resolution and sensitivity (LODs, 0.4–28.5 μg/kg). The proposed MISPE-CZE-MS/MS method successfully detected 9 AGs in honey samples and obtained good recovery (88.2–99.8%) and precision (RSDs lower than 8%). Hu et al. [151] established a novel approach for on-line enzyme assays for penicillinase using CE-integrated immobilized enzyme reactors (IMERs). The CE-IMER method was used to detect penicillin in pork samples, achieving good recovery (96.3–110.8%) and precision (RSDs, 1.5–3.1%). SPE in combination with a large-volume sample stacking (LVSS)-CE system has been described and applied to detect four TCs, providing sensitive, rapid, simple, and efficient on-line preconcentration of TC residues in milk samples [152]. The LODs obtained were 18.60–23.83 μg/L for the four analytes, with inter- and intraday repeatabilities <10%. To date, the CE method is widely used to detect various veterinary drugs in animal-derived foods [153,154,155,156,157].

### 4.5. Micellar Electrokinetic Capillary Chromatography (MEKC)

MEKC is a novel hybrid method that combines the separation principles of chromatography and electrophoresis. MEKC is an important form of CE and has become one of the most popular technologies for separating veterinary drugs due to its high separation power and ability to separate both ionic and neutral compounds [158]. In MEKC, an ionic surfactant such as sodium dodecyl sulfate (SDS) is added to the buffer to form micelles. The separated substances are distributed between the aqueous phase and the micellar phase and migrate in the capillary with electroosmotic flow, thereby achieving a separation effect [159]. Because of its simple operation, few sample pre-processing steps, and low instrumentation cost, this method was used in the detection of veterinary drug residues in animal-derived foods in recent years.

To date, the use of MEKC has been reported for detecting Q [159], LA [160], COC [161], AP [162], SA [163] and PCN [164] residues in poultry tissues, milk, and eggs. Kowalski et al. [160] established a MEKC method combined with a UVD for the detection of lincomycin and clindamycin in poultry tissue samples. In this study, a mixture of 10 mM sodium tetraborate decahydrate (pH 9.3) and 25 mM SDS was used as an ionic surfactant for MEKC. The LODs and LOQs of lincomycin and clindamycin were 13.2 and 18.5 μg/kg and 44.2 and 61.5 μg/kg, respectively. The recovery (97.5–109.5%) and precision (3.9–11.7%) of the proposed method show the successful application for lincomycin and clindamycin residues in poultry tissues. In addition, Belal et al. [161] developed a MEKC method by using a CE-DAD system for the detection of five COCs in chicken tissues. Under optimized separation conditions, the separation time of these analytes was less than 14 min. MEKC was performed in 50 mM tris buffer (pH 8.5) with 50 mM SDS and 15% ACN (*v/v*) at 28 kV and 200 nm. The LODs and LOQs were obtained for five COCs (65–172 and 183–493 μg/L), and the recovery and precision were obtained for chicken tissues (97.0–99.4% and 0.8–1.8%). Shao et al. [164] applied a novel analytical procedure developed for the simultaneous separation and detection of 6 PCNs in milk and eggs. For the analytical procedure, an on-line preconcentration and clean-up procedure was developed by using LVSS with polarity switching prior to MEKC or HPLC separation. Under the optimized conditions, the enrichment factors of 6 PCNs ranged from 150 to 601, and the LODs ranged from 0.16 to 0.26 μg/kg. This study used HPLC-UVD and MEKC methods to analyze the multi-residue content of 6 PCNs in milk and eggs. The HPLC-UVD and MEKC recoveries were all higher than 78.3% and 79.3%, respectively, and the RSDs were all lower than 4.1% and 5.2%, respectively. Compared with the HPLC-UVD method, the MEKC method achieves better separation. Some published ELISA, CE and MEKC methods for the detection of veterinary drugs in animal-derived foods are summarized in Table 5. 

## 5. Advanced Methods for Detection

With the continuous development of detection technology, advanced sensor-led detection technology has emerged, providing a fast, efficient, and cost-effective method for detecting veterinary drug residues in animal-derived foods. Traditional chromatographic techniques are used in combination with different detectors to provide sensitivity, specificity, and reliability for analysis, but the disadvantages are that they consume large amounts of organic reagents, are time consuming and expensive and are not suitable for a large number of screening samples. Advanced methods are used as alternative detection methods to detect veterinary drug residues in animal-derived foods, including methods involving electrochemical biosensors [21,165], piezoelectric biosensors [166], optical biosensors [167,168,169], and MIP biosensors [170,171]. The developed sensor analysis method not only has the advantages of simple operation, high speed and low cost but also provides satisfactory results in terms of specificity, sensitivity, and recovery in the detection of veterinary drugs in animal-derived foods.

To date, electrochemical biosensors have been used to detect CAP, TC, AMP and PCN in milk, honey, chicken, and beef [172,173,174,175,176]. Wang et al. [172] developed a green and efficient synthesis approach for chlorine-doped reduced graphene oxide (Cl-RGO) and constructed a highly selective electrochemical sensor for CAP based on Cl-RGO. The sensor has excellent reproducibility (102.4–103.5%) and storage stability and has been successfully used for CAP detection in milk samples. The application of MWCNTs and electropolymerized poly (L-glutamic acid) in electrochemical aptasensors has been used for the detection of TC in honey samples [173]. The developed method detected TC based on the direct electrodeposition of a polyglutamic acid (PGA)-MWCNT-glassy carbon electrode (GCE). The LOD obtained was as low as 3.7 × 10^−11^ mM for TC. This method uses one-step modification of the electrode surface, which decreases the time consumption of the modification process and provides good sensitivity. The research reported by Li et al. [176] is based on the manufacture of electrochemical biosensors with hybrid nanowire/nanoparticle arrays with various biomolecular receptors for simultaneous detection of PCN and TC in chicken and beef. This established method has obtained good recovery, precision (3.4–4.5%) and sensitivity (LOD, 10.5–15.2 μM) and has been successfully applied to the detection of PCN and TC in chicken and beef. Some published advanced methods, including methods that use electrochemical biosensors, piezoelectric biosensors, optical biosensors, MIP biosensors and other biosensors for the detection of veterinary drugs in animal-derived foods, are summarized in Table 6. Biosensors exhibit low reagent consumption and high sensitivity and save time and labor, and their application in the detection of veterinary drugs in animal-derived foods will become a trend.

## 6. Conclusions

Veterinary drug residues in animal-derived foods are an issue that warrants attention and are related to human health and safety. Different sample preparation techniques are suitable for different animal food matrices, each with its own advantages and disadvantages. However, compared with other sample preparation techniques, the ASE method has the advantages of full automation, low reagent consumption and batch processing of samples, so this method is worth promoting. Compared with LC and GC instruments, ELISA, CE and MEKC methods require relatively inexpensive testing equipment. For laboratories without LC and GC equipment, these three methods can be selected to screen and analyse veterinary drug residues. The LC-MS/MS method can be used to quantitatively and qualitatively analyse veterinary drug residues in animal-derived foods. This method has a wide range of applicability and does not require derivatization reactions, making it worthy of recommendation as a detection method. Considering the cost and time savings, advanced methods have been developed as alternative methods. Compared with analytical techniques, enzyme biosensors can provide better sensitivity and precision in the detection of veterinary drug residues in animal-derived foods and can detect lower concentrations. At present, biosensors that can detect veterinary drugs in animal-derived food are still limited, requiring researchers to continue to develop low cost, environmentally optimized biosensors.

## Figures and Tables

**Table 1:** Comparison of the efficacy of different cartridges for veterinary drugs in animal-derived foods.

Animal-Derived Food	Cartridge Type	Extraction Recovery (%)	LOD (μg/kg or μg/L)	Ref.
Eggs	CNWBOND LC-C18 (6 mL/150 mg)	71.7–102.7	0.16–0.52	[61]
Animal tissue, fish and honey	EVOLUTE ABN (3 mL/200 mg)	50.0–120.0	≥1.0	[62]
Milk and fish tissue	OASIS HLB (3 mL/60 mg)	–	15.0–200	[63]
Fish, shrimp and eel	OASIS PRIME HLB (6 mL/200 mg)	70.0–120.0	0.15–100	[64]
Dairy products	OASIS HLB (6 mL/200 mg)	67.3–106.9	0.006–0.3	[65]
Bovine muscle	OASIS HLB (6 mL/200 mg)	37.4–106.0	–	[66]
Milk	OASIS HLB (3 mL/60 mg)	68.0–118.0	0.01–5	[67]
Eggs	Hybrid SPE (1 mL/30 mg)	75.0–108.0	–	[68]

Note: “–” indicates not reported.

**Table 2:** Comparison of extraction methods for veterinary drugs (pros and cons).

Extraction Method	Pros	Cons
LLE	Simple, reliable, and widely applicable	Consumption of organic reagents and time consuming
SPE	Less time consuming than LLE Good purification effect and reproducibility	High cost of SPE cartridges Requires pre-treatment and toxic organic solvent
ASE	Low consumption of organic reagents Time saving Batch processing of samples Automated, fast, and convenient	High temperature and pressure, operation requires professional training
QuEChERS	Flexible and effective Simple instrumentation Low reagent consumption Wide scope of acidic and basic analytes	Low enrichment factors Low recovery of polar analytes
MSPD	Simple, efficient, and fast Low reagent consumption Wide scope of molecular structures and polar analytes	Relatively high degree of crushed samples

**Table 3:** Sample preparation techniques for the detection of veterinary drugs in animal-derived food samples.

Class of Veterinary Drugs	Animal-Derived Food	Sample Preparation Method	Ref.
MACs (12), LAs (2) and other contaminants (9)	Milk	LLE: 2 mL fresh milk sample + 15 mL ACN	[55]
MACs (10), Qs (15), TCs (5), SAs (27) and other contaminants (27)	Chicken muscle	LLE: 2 g sample + 5 mL EDTA-succinate + 10 mL ACN + 2 g sodium chloride	[57]
PCNs (2), APs (3), MACs (6), Qs (11), TCs (4), LAs (1), SAs (18), COCs (8) and other contaminants (62)	Milk powder, butter, fish tissue and eggs	LLE: 1 g sample + 2 mL 0.1% EDTA in H_2_O with 0.1% formic acid + 2 mL ACN + 2 mL MeOH	[58]
APs (4)	Eggs	LLE: 5 g sample + 1 mL ACN:water (30:70, *v/v*) + 20 mL ethyl acetate:ACN:ammonium hydroxide (49:49:2, *v/v*)	[60]
COCs (8)	Eggs	SPE: 2 g sample + 2 mL ultrapure water + 16 mL ACN: ethyl acetate (60:40, *v/v*):acetic acid (98:2, *v/v*) + CNWBOND C18 150 mg, elution 15 mL ethyl acetate	[61]
PCNs (7), APs (2), MACs (5), Qs (10), TCs (5), LAs (1), SAs (19), COCs (13) and other contaminants (81)	Milk and fish tissue	LLE: 1 g fish tissue sample + 2 mL 0.1% EDTA in H_2_O with 0.1% formic acid + 2 mL ACN + 2 mL MeOH SPE: 2 mL milk sample + 16 mL 5% trichloroacetic acid (TCA) in H_2_O:ACN (3:1, *v/v*) + 15% ammonia hydroxide (NH_3_·H_2_O) + Oasis HLB 60 mg, elution 6 mL MeOH	[63]
PCNs (6), MACs (6), AGs (6), SAs (14), COCs (12) and other contaminants (32)	Bovine muscle	SPE: 5 g sample + 10 mL ACN + 20 mL extraction solvent (consisting of 10 mM ammonium acetate, 0.4 mM EDTA, 1% NaCl and 2% TCA in H_2_O) + Oasis HLB 200 mg, elution 1 mL 10% formic acid and 3 mL ACN	[66]
MACs (3), Qs (8), TCs (4), LAs (1), SAs (8) and other contaminants (14)	Milk	SPE: 1 mL sample + 0.5 mL water + 3 mL ACN + 3 mL 0.1 mol/L phosphate buffer solution (PBS) + Oasis HLB 60 mg, elution 3 mL ACN:water (1:1, *v/v*)	[67]
AGs (1) and LAs (1)	Poultry eggs	ASE: 2 g sample + 4 g diatomaceous earth + 0.01 M KH_2_PO_4_ solution (a total solvent rinse of 50%), two cycles + 2 mL 0.2 M sodium dodecyl sulphonate (SDS) solution + Oasis PRiME HLB 60 mg, elution 6 mL MeOH	[12]
MACs (17) and other contaminants (1)	Swine and bovine tissues (muscle, kidney and liver)	ASE: 2 g sample + 12 g EDTA-treated sand + ACN: MeOH (1:1, *v/v*) (a total solvent rinse of 60%), two cycles + 5 mL MeOH	[71]
TCs (7)	Porcine, chicken and bovine (muscle and liver)	ASE: 2 g sample + 5 g EDTA-treated sand + ACN and 1 mM TCA (pH 4.0) (a total solvent rinse of 50%), two cycles	[72]
APs (4)	Poultry eggs	ASE: 5 g sample + 4 g diatomaceous earth + MeOH:NH_3_·H_2_O:ultrapure water (97:2:1, *v/v*) (a total solvent rinse of 40%), one cycle + 1 mL ACN + 10 mL hexane saturated with ACN + 5 mL ACN:water (4:6, *v/v*)	[11]
PCNs (2), APs (1), MACs (2), SAs (4) and other contaminants (5)	Milk	QuEChERS: 10 g sample + 100 μL acetic acid + 10 mL ACN + 4 g MgSO_4_ + 50 mg chitosan + 150 mg MgSO_4_	[75]
MACs (7), Qs (18), TCs (4), LAs (2), SAs (19) and other contaminants (40)	Royal jelly	QuEChERS: 1 g sample + 5 mL mixed solution of 0.1 M citric acid and 0.2 M Na_2_HPO_4_ (8:5, *v/v*, pH 4) + 20 mL 5% acetic acid in ACN + 2 g NaCl +2 g Na_2_SO_4_ + 200 mg NH_2_ sorbents	[76]
PCNs (2), APs (4), MACs (6), FQs (9), TCs (4), SAs (16) and other contaminants (9)	Flatfish, eel and shrimp	QuEChERS: 2 g sample + 1 mL 0.1 M EDTA in 50 mM ammonium acetate buffer solution (pH 4.0) + 9 mL 2 mM ammonium formate in water:ACN (1:4, *v/v*) + 250 mg PSA + 250 mg C18 sorbents	[77]
MACs (6), Qs (13), SAs (18) and other contaminants (18)	Porcine, bovine and ovine muscle	QuEChERS: 4 g sample + 16 mL 5% acetic acid in ACN + 2 g NaCl + 4 g Na_2_SO_4_ + 400 mg C18 sorbents	[80]
FQs (8), TCs (4) and SAs (8)	Pork	MSPD: 0.2 g sample + 0.15 g MMIP + 50 mg MMIP + 1 mL MeOH + 1 mL water + 3 mL MeOH:water (2:8, *v/v*) + 4 mL MeOH:acetic acid (9:1, v/v)	[84]
SAs (14)	Fish tissue	MSPD: 0.01 g sample + 0.02 g HLB + 2 mL ACN + 0.2 mL MeOH:water: NH_3_·H_2_O (50:49:1, *v/v/v*)	[85]
TCs (3)	Milk	MSPD: milk sample:sorbents (1:4, *m/m*) + 6 mL hexane + 6 mL 0.1 M citric acid aqueous solution:MeOH (1:9, *v/v*)	[88]
APs (3)	Fish muscle	MSPD: 2 g sample + 3 g C18 sorbents + 8 mL hexane + 10 mL ACN:water (1:1, *v/v*) + 6 mL ethyl acetate	[17]
APs (4), MACs (18), Qs (21), TCs (7), LAs (3), SAs (24) and other contaminants (43)	Edible muscles, eggs and milk	UAE: 2 g sample + 10 mL ACN:water (9:1, *v/v*) + 10 min UAE + 5 mL water + Oasis HLB 500 mg, elution 5 mL formic acid:MeOH (5:95, *v/v*) and 5 mL ethyl acetate	[18]
COCs (9)	Eggs	GPC: 2 g sample + 5 g anhydrous sodium sulfate + 10 mL ethyl acetate + online gel permeation chromatographic cleanup	[92]
PCNs (8), MACs (5), AGs (1), Qs (7), TCs (4), SAs (6) and other contaminants (9)	Honey	TFC: 1 g sample + 1 mL 0.1 M Na_2_EDTA (pH 4) + Millex-GN nylon filter (0.20 μm) + online sample extraction by TFC procedure	[93]
APs (3)	Milk	FPSE: FPSE media in 1 mL Cameo (1:1, *v/v*) + 0.5 g sample, kept for 30 min + remove the FPSE media from the extraction via and insert it into backextraction containing 0.5 mL MeOH for 10 min	[94]
TCs (2)	Chicken, fish and milk	SPME: 5 mL or 5 g sample + 20 mL Na_2_EDTA-McIlvaine extract buffer + a homemade SPME device, elution 2 mL ACN:formic acid (2:1, *v/v*)	[95]
SAs (5)	Shrimp	SLE: 0.5 g sample + 3 mL MeOH:ACN (50:50 *v/v*) + 0.5 mL MeOH:0.1% acetic acid aqueous solution (40:60 *v/v*) + the supernatant was transferred to the falcon tube + 0.5 mL MeOH:0.1% acetic acid aqueous solution (40:60 *v/v*)	[96]
TCs (4) and Qs (5)	Lamb and chicken tissues, fish, honey, and milk	LPME: 5 g lamb and chicken tissues and fish samples + 15 mL ACN + 5 g sodium sulfate + 19 aqueous solution mL (pH 12.0) 5 g honey sample + 5 mL 2 mol/L HCl + 10 mL NaOH solution (2 mol/L) 20 mL milk sample + 10 mL 0.5 mol/L K_3_[Fe(CN)_6_]·3H_2_O solution + 10 mL 2 mol/L Zn(CH_3_COO)_2_·2H_2_O	[97]
TCs (6)	Beef	DLLME: 1 g sample + 6 mL water:ACN (5:1, *v/v*) + 300 mg magnesium sulfate anhydrous + 150 mg sodium chloride + 50 mg trisodium citrate dehydrate + sodium hydroxide solution and formic acid, adjust to pH 7 + 1 mL methanol + 200 μL dichloromethane + 100 μL water	[98]

**Table 4:** Overview of published LC and GC methods for the analysis of veterinary drugs in animal-derived foods.

Class of Veterinary Drugs	Animal-Derived Food	Sample Preparation Method	Detection Method	Recovery (%)	RSD (%)	LOD (μg/kg or μg/L)	LOQ (μg/kg or μg/L)	Ref.
SAs (7)	Cattle meats	LLE	HPLC–FLD	44.6–81.0	2.7–4.9	8–15	13–25	[99]
APs (3)	Poultry eggs	ASE	UPLC–FLD	80.1–98.6	1.2–4.3	1.8–4.9	4.3–11.7	[100]
APs (3)	Milk	FPSE	HPLC–DAD	92.3–106.0	1.0–10.7	–	–	[94]
SAs (5)	Shrimp	SLE	HPLC–DAD	90.2–109.0	1.5–14.4	15	50	[96]
TCs (4) and Qs (5)	Lamb and chicken tissues, fish, honey, and milk	LPME	HPLC–DAD	25.5–82.6	3.4–10.7	0.5–20	1.25–50	[97]
FQs (2), TCs (1) and SAs (2)	Porcine tissues	MSPD	HPLC–DAD	80.6–99.2	0.3–6.1	2–10	7–34	[101]
PCNs (6) and APs (3)	Gilthead seabream tissues	SPE	HPLC–DAD	95.6–104.0	0.3–6.7	11.0–20.4	33.2–61.7	[102]
COCs (2)	Cattle and chicken muscle	SPE	HPLC–UVD	78.5–107.1	2.2–10.9	40–130	130–420	[103]
SAs (4)	Chicken muscle	LLE	HPLC–UVD	70.0–84.0	8.0–13.0	0.14–6.53	0.42–19.6	[104]
LAs (1)	Milk	SPE	HPLC–UVD	80.0–89.0	0.8–4.7	20	80	[105]
PCNs (2)	Eggs, chicken and bovine tissues	LLE	HPLC–UVD	95.5–102.3	0.4–1.2	500–1300	1700–4500	[106]
TCs (3)	Eggs, milk and milk powder	SPE	HPLC–UVD	85.3–98.3	1.9–5.3	0.76–1.13	2.53–3.77	[107]
TCs (4)	Milk and eggs	SPE	HPLC–UVD	84.2–98.6	1.4–5.9	1.03–2.67	3.46–8.97	[108]
SAs (4)	Chicken meat	SPE	HPLC–UVD	92.0–106.0	3.8–6.7	0.5–150	–	[109]
SAs (15)	Milk, pork, beef and mutton tissues	LLE	HPLC–UVD	81.5–95.3	0.8–7.4	6.5–11.0	–	[110]
MACs (7), Qs (18), TCs (4), LAs (2), SAs (19) and other contaminants (40)	Royal jelly	QuEChERS	UPLC–QTOF– MS	70.2–120.1	1.8–9.9	0.06–6.0	0.21–20	[76]
PCNs (2), APs (1) and TCs (2)	Milk	QuEChERS	LC–TOF–MS	83.0–92.0	1.1–8.8	0.0075–1.92	0.025–6.39	[78]
PCNs (8), MACs (5), AGs (1), Qs (7), TCs (4), SAs (6) and other contaminants (9)	Honey	TFC	UPLC– Orbitrap–MS	68.0–121.0	1.0–25.0	0.1–50	5–50	[93]
SAs (7) and other contaminants (6)	Shrimp	QuEChERS	LC–TOF–MS	58.0–133.0	4.7–14.9	0.06–7	–	[79]
PCNs (7), APs (2), MACs (8), AGs (15), FQs (17), TCs (5), SAs (26) and other contaminants (45)	Milk	QuEChERS or SLE or SPE	UPLC– Orbitrap–MS	12.4–146.2	0.9–54.8	≤1.0	≤ 5.0	[120]
SAs (7)	Eggs	SPE	LC–MS/MS	73.8–96.2	2.9–8.3	1.4–2.8	4.7–9.2	[114]
PCNs (4)	Chicken tissues	LLE	UPLC–MS/MS	84.1–108.1	1.3–16.4	0.01–1.36	0.05–5.44	[115]
APs (4)	Poultry eggs	ASE	LC–MS/MS	88.3–107.0	1.5–3.9	0.04–0.5	0.1–1.5	[11]
COCs (8)	Eggs	SPE	LC–MS/MS	71.7–102.7	2.6–15.3	0.16–0.52	0.81–1.73	[61]
MACs (7)	Pork	SPE	LC–MS/MS	68.6–95.5	0.5–7.6	0.2–0.5	0.5–2.0	[121]
AGs (15)	Pig, chicken and cattle tissues, milk, and eggs	SPE	LC–MS/MS	71.4–93.9	2.0–13.0	5 –10	5 –20	[122]
Qs (9)	Ovine, chicken and porcine tissues, eggs, milk and fish	SPE	HPLC–FLD LC–MS/MS	50.0–128.0	<30.0	–	–	[123]
TCs (6)	Beef	DLLME	LC–MS/MS	80.0–105.0	2.0–7.0	2.2–3.6	7.4–11.5	[98]
APs (3)	Fish muscle	MSPD	UPLC–MS/MS	84.2–99.8	5.6–11.4	0.02–0.06	0.11–0.16	[17]
MACs (5) and LAs (2)	Meat and milk	ASE	LC–MS/MS	70.0–93.0	2.7–11.3	3–10	10–30	[124]
AGs (1) and LAs (1)	Animal tissues	ASE	GC–NPD	73.0–99.0	<17.0	8.1–12.1	16.4–21.4	[117]
AGs (1) and LAs (1)	Animal tissues	ASE	GC–MS	70.0–93.0	<21.0	1.9–3.1	4.1–5.6	[117]
APs (4)	Poultry and porcine tissues	SPE	GC–MS	78.5–105.5	6.4–16.8	0.1–0.5	0.25–2	[125]
APs (2)	Fish tissues	SPE	GC–MS	84.1–100.9	1.3–2. 7	1.64–9.3	4.9–29.4	[126]
APs (1) and other contaminants (8)	Fish	QuEChERS–GPC	GC–MS	63.5–90.2	3.6–15.4	0.3–1.0	–	[127]
AGs (1) and LAs (1)	Poultry eggs	ASE–SPE	GC–MS/MS	80.0–95.7	1.0–3.4	2.3–4.3	5.6–9.5	[12]
COCs (1)	Chicken and pig tissues	ASE–SPE	GC–MS/MS	77.5–96.3	1.6–6.6	1.4–1.6	4.8–5.2	[119]
COCs (2)	Chicken tissues	ASE–SPE	GC–MS/MS	82.0–94.3	1.7–5.4	0.8–2.5	2.7–8.0	[128]
COCs (2)	Eggs	ASE	GC–MS/MS	82.7–87.5	1.7–4.6	0.8–2.8	3.0–10.0	[129]

Note: “–” indicates not reported.

**Table 5:** Overview of published ELISA, CE and MEKC methods for the analysis of veterinary drugs in animal-derived foods.

Veterinary Drug	Class	Animal-Derived Food	Sample Preparation Method	Detection Method	Recovery (%)	RSD (%)	LOD (μg/kg or μg/L)	LOQ (μg/kg or μg/L)	Ref.
FF and TAP	APs	Animal tissues	LLE	ic-ELISA	80.6–105.5	3.5–14.1	0.07–0.14	–	[130]
LIN	LAs	Milk and honey	LLE	ic-ELISA	84.6–115.6	1.7–25.4	2.1	–	[131]
ERY	MACs	Milk	LLE	ic-ELISA	76.9–85.7	5.1–11.3	0.3	–	[132]
SAL	COCs	Chicken tissues	LLE	ic-ELISA	85.7–99.3	1.6–6.6	18–22	–	[133]
20 SAs	SAs	Animal tissues	LLE	ic-ELISA	70.6–121.0	0.8–24.1	1.5–22.3	–	[134]
4 TCs	TCs	Animal meat and milk	LLE	ELISA	71.9–100.0	<10.0	3.7–9	9–27	[135]
13 FQs and 22 SAs	FQs and SAs	Milk	MSPD	DC-ELISA	67.0–105.0	4.8–16.4	2.4–5.8	–	[136]
NEO	AGs	Animal tissues, eggs and milk	LLE	ELISA	65.8–122.8	5.9–28.6	5.7–29.3	11.4–59.7	[137]
6 PCNs and 4 TCs	PCNs and TCs	Milk	LLE	ic-ELISA	80.8–99.4	3.0–12.7	0.4–3.7	–	[138]
KAN and STR	AGs	Milk	LLE	dot-ELISA	84.2–124.5	4.5–12.4	0.09–1.37	0.38–38.66	[139]
4 SAs	SAs	Milk	MSPE	CE–UVD	62.7–104.8	3.9–10.2	0.89–2.31	–	[143]
SDZ, SMR and SMZ	SAs	Milk	SPME	CE–LIF	91.1–94.6	0.9–1.1	0.25‒0.47	0.78‒1.54	[144]
AZI, TIL, ACE and ROX	MACs	Egg	LLE	CE–ECL	89.3–107.5	1.3–5.6	1.3‒70 nmol/L	93–2100 nmol/L	[145]
7 Qs	Qs	Milk	MSPE	CE–DAD	74.0–98.0	1.0–9.9	9‒12	–	[146]
8 Qs	Qs	Milk	MISPE	CE–MS/MS	70.0–102.3	3.0–12.0	1.0–1.4	3.2–4.7	[147]
9 AGs	AGs	Honey	MISPE	CE–MS/MS	88.2–99.8	2.4–6.8	0.4–28.5	1.4–94.8	[148]
SDD, SDZ and STZ	SAs	Milk, pork and chicken meat	SPE	CE–CL	79.5–112.4	2.1–2.8	0.65–3.14	–	[149]
6 SAs	SAs	Milk, pork and egg	LLE	PAEKI–CZE	89.0–113.0	1.6–8.4	1.8–63.8	6.1–182.6	[150]
PCN	PCNs	Pork	LLE	CE–IMERs	96.3–110.8	1.5–3.1	–	–	[151]
CT, DT, OT and TC	TCs	Milk	SPE	LVSS-CE	–	1.7–9.7	18.6–23.83	–	[152]
4 Qs and 3 SAs	Qs and SAs	Aquatic product	ASE	LVSS-CE	84.3–95.7	1.1–4.7	13–35	40–100	[153]
OT	TCs	Milk	SPME	CE–DAD	89.9	2.25	70	–	[154]
PCN G and PCN acid	PCNs	Milk	LLE	CZE	89.2–96.8	3.1–7.3	10–500	40–1700	[155]
8 TCs and 7 Qs	TCs and Qs	Milk	LLE	CZE–QTOF-MS	72.6–105.8	2.1–10.5	0.5–2.9	1.6–9.7	[156]
TIL and TYL	MACs	Chicken fat	RUSAEME	CE–DAD	73.0–117.0	0.7–12.4	5.2–18.9	17.4–55.0	[157]
LIN and CLI	LAs	Poultry tissues	SPE	MEKC–UVD	97.5–109.5	3.9–11.7	13.2–18.5	44.2–61.5	[160]
5 COCs	COCs	Chicken Tissues	LLE	MEKC–DAD	97.0–99.4	0.8–1.8	65–172	183–493	[161]
CIP, ENR, CAP and FF	FQs and APs	Milk	SPE	MEKC–DAD	80.0–109.0	0.1–4.8	6.8–13.9	–	[162]
7 SAs and 3 APs	SAs and APs	Poultry tissues	SPE	MEKC–UVD	86.4–109.4	3.1–14.9	1.3–7.8	4.5–26.1	[163]
6 PCNs	PCNs	Milk and egg	LLE	LVSS-MEKC–UVD	79.3–103.6	2.0–5.2	0.16–0.26	2	[164]

Abbreviations: lincomycin, LIN; erythromycin, ERY; salinomycin, SAL; dual-colorimetric ELISA, DC-ELISA; neomycin, NEO; kanamycin, KAN; streptomycin, STR; magnetic solid-phase extraction, MSPE; sulphadiazine, SDZ; sulphamerazine, SMR; sulphamethazine, SMZ; azithromycin, AZI; tilmicosin, TIL; acetylspiramycin, ACE; roxithromycin, ROX, sulphadimidine, SDD; sulphathiazole, STZ; penicillin, PCN; chlortetracycline, CT; doxycycline, DT; oxytetracycline, OT; tetracycline, TC; tylosin, TYL; reverse ultrasound-assisted emulsification-microextraction, RUSAEME; clindamycin, CLI; ciprofloxacin, CIP; enrofloxacin. ENR. Note: “–” indicates not reported.

**Table 6:** Overview of published advanced methods for the analysis of veterinary drugs in animal-derived foods.

Veterinary Drug	Class	Animal-Derived Food	Detection Method	Recovery (%)	LOD (μM or μg/L)	Ref.
CAP	APs	Milk	Electrochemical sensor	102.4–103.5	1	[172]
TC	TCs	Honey	Electrochemical aptasensor	94.0–95.0	3.7 × 10^−11^	[173]
AMP	PCNs	Milk	Electrochemical biosensor	95.0–98.1	1.0×10^−3^	[174]
PCN	PCNs	Milk	Electrochemical aptasensor	96.0–105.4	0.057	[175]
PCN and TC	PCNs and TCs	Chicken and beef	Electrochemical biosensor	–	10.5–15.2	[176]
STZ	SAs	Honey	Piezoelectric immunosensor	100.0–113.0	0.1 μg/kg	[166]
CAP, SDZ and NEO	APs, SAs and AGs	Milk	Optical fiber-mediated immunosensor	85.0−109.4	0.00286–30	[177]
CAP	APs	Porcine muscle, honey, milk and prawn	MIP biosensor	87.0−103.0	7 × 10^−5^	[178]
CAP	APs	Milk	MIP biosensor	96.0−105.0	3×10^−7^	[179]
NEO and KAN	AGs	Dairy products	Ellipsometric sensor	96.8−106.3	0.048–0.22	[180]
PCN	PCNs	Milk	Single layer and bilayer potentiometric biosensors	102.0−124.0	0.3	[181]

Note: “–” indicates not reported.

## Data Availability

Data supporting reported results can be obtained from any masthead.
